# Analysis of urine cell-free DNA in bladder cancer diagnosis by emerging bioactive technologies and materials

**DOI:** 10.3389/fbioe.2024.1458362

**Published:** 2024-09-04

**Authors:** Fei-Fei Huang, Xiao-Fei Di, Mo-Han Bai

**Affiliations:** School of Medicine, South China University of Technology, Guangzhou, Guangdong, China

**Keywords:** bladder cancer, urinary cell-free DNA, UcfDNA, noninvasive diagnosis, molecular characterization

## Abstract

Urinary cell-free DNA (UcfDNA) is gaining recognition as an important biomarker for diagnosing bladder cancer. UcfDNA contains tumor derived DNA sequences, making it a viable candidate for non-invasive early detection, diagnosis, and surveillance of bladder cancer. The quantification and qualification of UcfDNA have demonstrated high sensitivity and specificity in the molecular characterization of bladder cancer. However, precise analysis of UcfDNA for clinical bladder cancer diagnosis remains challenging. This review summarizes the history of UcfDNA discovery, its biological properties, and the quantitative and qualitative evaluations of UcfDNA for its clinical significance and utility in bladder cancer patients, emphasizing the critical role of UcfDNA in bladder cancer diagnosis. Emerging bioactive technologies and materials currently offer promising tools for multiple UcfDNA analysis, aiming to achieve more precise and efficient capture of UcfDNA, thereby significantly enhancing diagnostic accuracy. This review also highlights breakthroughs in detection technologies and substrates with the potential to revolutionize bladder cancer diagnosis in clinic.

## 1 Introduction

Data published by GLOBOCAN in 2020 ranks bladder cancer (BC) as the 10th most common cancer globally and the ninth leading cause of cancer-related mortality (Richters et al., 2020; Sung et al., 2021). Given the significant morbidity and mortality associated with BC, early detection of primary bladder carcinoma is crucial for effective patient management. Staging and grading not only aid in classifying patient cohorts but also essential for establishing prognostic outlooks (Koguchi et al., 2022). Despite being standard diagnostic practices, cystoscopy and urinary cytology suffer from high false-negative rates, hindering early disease identification. Although cystoscopy has shown high sensitivity, it is an invasive procedure heavily dependent on the operator’s technical expertise, and it may lead to complications, such as infection and urinary retention. Conversely, urinary cytology, although non-invasive and sensitive, is limited by its moderate specificity and dependence on sampling methods.

Since the early 2010s, researchers have explored liquid biopsy technology to revolutionize diagnostics. Utilizing biomarkers such as circulating tumor cells (CTCs) and nucleic acids, including cell-free DNA (cfDNA) and microRNAs (miRNAs), this technique shows promise for diagnosing various malignancies. These biomarkers are detectable in bodily fluids and vesicular emissions from neoplastic cells (Fais et al., 2016), with CTCs found in blood and both cfDNA and miRNAs detectable in blood and urine (Oliveira et al., 2020). For urothelial carcinoma, recent advances have focused on examining urinary cell-free DNA (UcfDNA) and miRNAs to facilitate early diagnosis. As a direct derivative of urothelium, UcfDNA shows significant potential as a biomarker for early detection, ongoing medical surveillance, and monitoring of postoperative recurrence in BC (Dawson et al., 2013; Tie et al., 2016).

Several studies have demonstrated that both qualitative and quantitative assessments of UcfDNA exhibit high sensitivity and specificity for BC diagnosis, underscoring the substantial potential of UcfDNA analysis. With advancements in biotechnology, growing array of new technologies and materials featuring high separation purity, low cost, automation, and rapid detection capabilities is emerging. These innovations have shown significant capacity for effectively capturing UcfDNA, thereby enhancing the speed and accuracy of diagnostic tests for bladder cancer. Accordingly, this review comprehensively examines multiple roles of UcfDNA in bladder cancer diagnosis, with a particular focus on the advanced technologies and materials employed in UcfDNA detection.

## 2 Biological characteristics of UcfDNA

**FIGURE 1:**
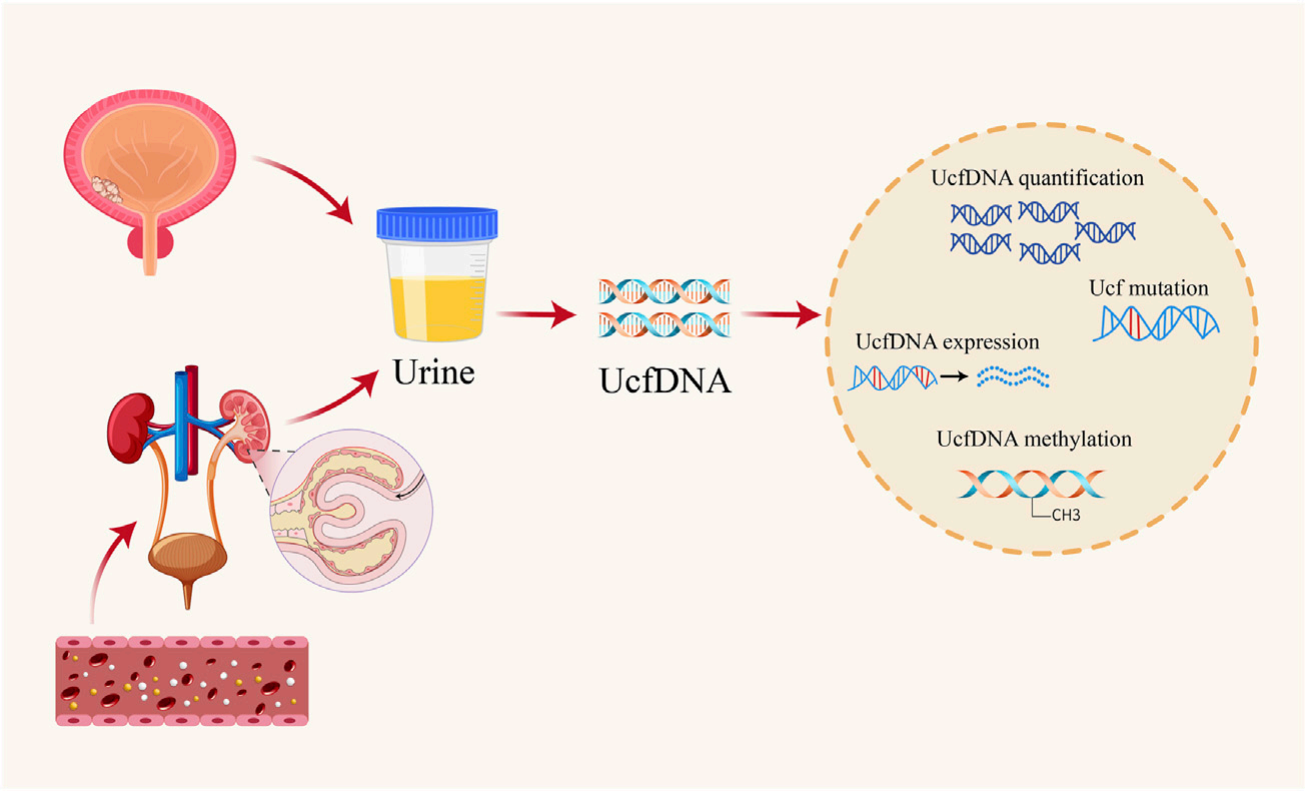
Origins of UcfDNA and common testing contents.

Urinary cell-free DNA (UcfDNA), consisting of unbound DNA fragments in urine, primarily originates from two sources: shedding from the genitourinary tract epithelium and renal filtration (Figure 1). DNA released during apoptosis or necrosis within urinary organs is predominantly high molecular weight, often exceeding 1,000 base pairs. In contrast to blood-derived DNA, which is bound to proteins and unable to pass through the glomerular filter due to its size, only small DNA fragments, ranging from 40 to 250 base pairs, are filtered into urine (Udomruk et al., 2021). Methodologies used for UcfDNA isolation have identified distinct high- and low-molecular-weight fractions (Su et al., 2004).

Under neoplastic conditions, UcfDNA profiles change significantly as tumor cells release cfDNA into urine, primarily from necrotic and apoptotic cancer cells, typically in double-stranded structures (Corcoran and Chabner, 2018). This cfDNA source allows analysis of genetic anomalies indicative of genomic diversity in bladder cancer. Furthermore, cfDNA facilitates exploration of molecular variations associated with treatment resistance, often marked by simultaneous growth of multiple pharmacoresistant subclones within a single lesion or across metastatic sites (Russo et al., 2016; Goyal et al., 2017; Hazar-Rethinam et al., 2018; Diaz et al., 2012). For example, using molecular biopsies of focal tumor cells and cfDNA analysis to examine and monitor colorectal cancer patients, the K57T MEK1 mutation was found to be a novel mechanism of acquired resistance in patients with progressive liver metastases after long-term response to cetuximab, whereas the lesions receded with treatment of panitumumab and the MEK inhibitor trametinib and the level of the mutant MEK1 in cfDNA declined with treatment, confirming the role of cfDNA in exploring the molecular variants associated with drug resistance (Russo et al., 2016). Other investigators have reported the molecular basis of acquired BGJ398 resistance in patients with cholangiocarcinoma by comprehensive genomic characterization of primary tumors, cfDNA, and metastases, and later by sequential analyses of cfDNA that identified multiple recurrent point mutations in the structural domain of the FGFR2 kinase at the time of progression, which underpinned inter- and intra-lesional heterogeneity detected in biopsies of late-progressing lesions (Goyal et al., 2017).

Recognized for its specificity and sensitivity, cfDNA has emerged as a valuable tumor marker in liquid biopsies, noted for its minimally invasive nature and comprehensive reflection of tumor heterogeneity, leading non-invasive diagnostic strategies (Liu and Wang, 2022). Blood-derived cfDNA is well-regarded for its high stability (Igari et al., 2022). However, UcfDNA offers distinct advantages for diagnosing bladder cancer, including direct tumor contact, simple and non-invasive collection unaffected by technical proficiency, and lower protein interference (Satyal et al., 2019). Despite these benefits, susceptibility to DNase-induced degradation in urine must be addressed to maintain the diagnostic utility of UcfDNA.

## 3 UcfDNA quantification as a biomarker for bladder cancer

### 3.1 Quantitative UcfDNA detection methods

Quantitative detection of cfDNA is widely recognized for its extensive potential in oncology, employing diverse methodologies. The analytical process begins with urine sample collection, followed by cfDNA extraction and quantification. Collecting urine samples is straightforward, requiring only sterile containers and centrifugation to separate the supernatant (Salvi et al., 2016). After collection, cfDNA can be isolated using specific DNA extraction kits or adsorption columns designed for this purpose. The principles of extraction vary: some kits utilize organic solvents, while others rely on selective adhesion to silica-based materials or magnetic beads. Traditional laboratory methods such as the phenol-chloroform method (PCI-glycogen) and the sodium iodide method (NaI) also remain viable options for cfDNA isolation (Fong et al., 2009).

Following extraction, various techniques are available for cfDNA quantification, including spectrophotometry (NanoDrop, GeneQuant Pro), fluorescence quantification (Qubit, PicoGreen), nucleic acid electrophoresis, and Polymerase Chain Reaction (PCR) amplification. These methodologies enable precise determination of cfDNA concentrations by assessing DNA content and amplification efficiency (Ralla et al., 2014). It is important to acknowledge that although these methods effectively quantify cfDNA, each presents unique advantages and limitations.

#### 3.1.1 Spectrophotometry

Spectrophotometry is based on the principle that DNA absorbs light at specific wavelengths proportional to its concentration. To determine the concentration of a cfDNA sample, a spectrophotometer measures the absorption intensity. This technique is valued for its high sensitivity, simplicity, speed, affordability, and widespread use in the scientific community. However, external factors can affect the accuracy of these measurements highlighting the need for improved precision.

#### 3.1.2 Fluorescence quantification method

Fluorescence quantification uses fluorescent dyes to detect the concentration of specific target molecules. These fluorescent dyes bind to DNA molecules and then emit fluorescent signals and these emitted signals are then measured using advanced fluorescence detection techniques to accurately quantify the DNA concentrations. Commonly used dyes include Propidium Iodide (PI), DAPI, EB, SYBR Green, *etc.* They can be embedded between bases to bind to DNA, and then fluoresce in different colors when illuminated by light sources of different wavelengths. Due to its remarkable sensitivity and specificity, this method is particularly effective for assessing low concentrations of nucleic acids which is consistent with the properties of cfDNA. However, the method has some limitations: it is costly, the efficiency of fluorescent labeling requires refinement, and variations in DNA extraction and dilution practices can affect the quantification process, as noted in the literature (Schirripa Spagnolo et al., 2023; Di Meo et al., 2017).

#### 3.1.3 Polymerase chain reaction

Polymerase chain reaction (PCR) invloves cyclical processes, including high-temperature denaturation, low-temperature annealing, and moderate-temperature extension reactions. These cycles exponentially amplify the target DNA, facilitating the quantification of the original DNA concentrations in samples based on the degree of amplification achieved. Advances in PCR have led to enhanced methods, notably real-time fluorescence quantitative PCR (qPCR). This technique integrates fluorescent dyes or probes within the PCR system, enabling real-time monitoring of amplification products *via* fluorescence signal detection. Analytical quantification is performed using CT values and standard curves. Two primary approaches within qPCR are prominent: the fluorescent probe method, typically employing hydrolysis probes, and the fluorescent dye method, commonly utilizing SYBR GREEN dye. The versatility of qPCR stems from its reliance on selective DNA templates.

Digital PCR (dPCR) is a more recent advancement, based on the Poisson distribution principle. This method involves distributing DNA or RNA samples across numerous microscopic reaction units, where single-molecule templates undergo amplification, followed by fluorescence detection and statistical analysis for absolute quantification (Zhou et al., 2019; Luo et al., 2024). Unlike traditional techniques, dPCR operates independently of standard curves and external standards, directly assessing the concentration of nucleic acids in samples, thereby enhancing efficiency over conventional PCR methodologies. Despite these advancements, PCR technologies remain prone to contamination and false-positive results, underscoring the need for further methodological refinement.

#### 3.1.4 Nucleic acid electrophoresis

The principle of DNA content measurement by nucleic acid electrophoresis is mainly based on the agarose gel electrophoresis technique. The agarose gel is placed in an electric field, and then DNA molecules are added to make them migrate in the same direction under the action of the electric field. DNA molecules of different molecular weights, different charges and different conformations migrate different distances in the electric field. Finally, the distance traveled by the target DNA under the action of the electric field is compared with the distance traveled by the standard DNA in the electric field to obtain the content of the target DNA molecules. This technique can be used to determine the molecular weight of cell-free DNA (cfDNA) and is straightforward and demonstrates high stability and sensitivity. However, it requires high quality samples and sophisticated handling skills.

### 3.2 Quantification of UcfDNA in bladder cancer diagnosis

The potential of UcfDNA quantification as a biomarker for bladder cancer has been explored through various methodologies (Table 1). One study reported no significant difference in the median UcfDNA levels between bladder cancer patients and healthy subjects. Utilizing four different DNA quantification methods—GeneQuant Pro, Quant-iT DNA High Sensitivity Detection Kit, Real-Time PCR, and NanoDrop 1,000— the study found that the area under the Receiver Operating Characteristic (ROC) curve for all methods was approximately 0.5, indicating no significant association with tumor size or presence (Zancan et al., 2009).

**TABLE 1:** Common techniques for detecting UcfDNA and its diagnostic value in bladder cancer.

Techniques	Markers	Methods	Results	Ref.
UcfDNA quantification	- UcfDNA quantification	- GeneQuant Pro - Quant-iT™ DNA assay kit - qPCR - NanoDrop	- Median UcfDNA quantification does not differ statistically between BC patients and healthy controls	Zancan et al. (2009)
	- UcfDNA/UCr concentration	- PicoGreen −400-bp qPCR	- Mean concentrations in BC patients exceed controls significantly - No significant difference is found between different stages and grades	Chang et al. (2007)
	- Total amounts of UcfDNA	- qPCR	- Total amounts of UcfDNA distinguishes BC patients from control - Benign group differs from pT1-T4 cancer group, aligns with pTa group	Brisuda et al. (2016)
UcfDNA mutation	- TERT (C228T/C250T)	- UroMuTERT	- Mutations exhibit high sensitivity and specificity in UC detection - Mutations surpass urinary cytology in detecting early, low-grade UC	Avogbe et al. (2019)
		- NGS	- Urine mutations enhance and eclipse voided cytology - CfDNA analysis offers no advantage over urine sediment DNA diagnostically	Stasik et al. (2019)
	- ADGRG6 enhancer mutation	- ARMS‐qPCR	- The sensitivity of the mutation is 83.3% and the specificity is 98.4%	Tan et al. (2023)
	- TERT promoter mutation and GPR126 enhancer mutation	- ddPCR	- Combined urine non-coding mutation analysis enables sensitive, non-invasive UBC detection	Jain et al. (2023)
	- TERT (C228T/C250T), FGFR3 (S249C) mutations		- TERT/FGFR3 UcfDNA analysis may serve as a diagnostic marker and staging factor	Hayashi et al. (2019)
	- TERT, FGFR3 mutations		- Drop dPCR of UcfDNA is a simple and promising UBC assay	Hayashi et al. (2020)
	- 5-gene panel (TERT, TP53, FGFR3, PIK3CA and ARID1A)	- PredicineCARE NGS	- 90% UBC detection with 5 genes; 90% of patients carry a mutation in those genes	Zhang et al. (2021)
	- 5-gene panel (TERT, FGFR3, TP53, PIK3CA and KRAS) - 7-gene panel (TERT, FGFR3, TP53, HRAS, PIK3CA, KRAS and ERBB2)	- NGS	- The AUC of the 5-gene panel is 0.94, and 7-gene panel is 0.91	Ou et al. (2020)
UcfDNA expression	- IQGAP3	- PicoGreen - RiboGreen	- IQGAP3 levels in BC are higher than in normals or hematuria - IQGAP3 expression rises with tumor grade - IQGAP3 differentiates MIBC from NMIBC	Kim et al. (2018)
	- IQGAP/BMP4 - IQGAP3/FAM107A	- qPCR	- UcfDNA biomarkers are markedly elevated in BC than in hematuria patients	Xu et al. (2019)
	- TopoⅡA		- TopoIIA expression in BC exceeds controls - TopoIIA UcfDNA higher in MIBC than NMIBC	Kim et al. (2016)
UcfDNA epigenetics	- 4-gene panel (HOXA9, PCDH17, POU4F2, and ONECUT2)	-MS-HRM PCR	- Urinary biomarkers may distinguish BC from benign hematuria diseases	Wu et al. (2020)
	- 7-gene panel (HOXA9, ONECUT2, PCDH17, PENK, TWIST1, VIM and ZNF154)	- MSP	- 7-gene panel predicts BC accurately in haematuria patients	Zhang et al. (2019)
	- TWIST1 and NID2		- Methylated TWIST1/NID2 in urine sediments offers highly sensitive, specific BC detection	Renard et al. (2010)
	- 3-gene panel (GDF15, TMEFF and VIM)		- Epigenetics differentiates BC from healthy and renal/prostate cancer patients	Costa et al. (2010)
	- RBBP8	- qMSP	- RBBP8 methylation in urine correlates with high-grade tumors	Mijnes et al. (2018)
	- DMRTA2		- mDMRTA2 methylation in urine is highly effective for primary and recurring BC detection	Deng et al. (2022)

NGS, next-generation sequencing; ddPCR, droplet digital PCR; qPCR, real-time quantitative PCR; ARMS-qPCR, amplification refractory mutation system combined with quantitative real-time PCR; MSP, methylation-specific polymerase chain reaction; qMSP, Real-time quantitative methylation-specific PCR (qPCR).

Conversely, other studies have suggested that UcfDNA quantification may more effectively differentiate between individuals with bladder cancer and healthy controls. Chang et al. used two techniques: PicoGreen and 400 bp UcfDNA normalized to urinary creatinine (Ucf-DNA/Ucr). Due to variability in urinary creatinine from differences in glomerular filtration and metabolism, the latter method provides an adjusted measure of UcfDNA concentration. Both assays detected higher mean UcfDNA levels in patient compared to controls, but the discriminative power between various grades of bladder malignancy was not apparent. However, the 400 bp Ucf-DNA/Ucr technique demonstrated superior reliability in cfDNA detection, achieving an ROC curve area of 0.805 with a sensitivity of 86.1% and a specificity of 72.0%. In contrast, the ROC area for PicoGreen was only 0.571 (Brisuda et al., 2016; Chang et al., 2007).

Expanding the scope, Brisuda et al. (2016) included patients with benign urological conditions to further elucidate tumor staging, using real-time PCR to quantify UcfDNA. Their finding showed that the total UcfDNA assessment could differentiate between patients with bladder cancer and those without, with an ROC area of 0.725 and positive and negative predictive values of 90% and 45%, respectively. The cohort with benign urinary diseases did not show a marked distinction from those with bladder neoplasms, likely due to the influence of the pTa group. A significant difference was observed when comparing the benign group with pT1-T4 group, but not within the pTa subset, suggesting its similarity to benign urological conditions in terms of UcfDNA concentrations. The prevalence of pTa tumors among undiagnosed bladder cancers may lead to suboptimal detection rates. Additionally, the timing of urination affected UcfDNA evaluation, with variations in concentration and volume during successive morning voids. Thus, the second morning urine specimen, characterized by lower concentration and reduced cellular lysis, was selected for analysis (Brisuda et al., 2016).

Taken together, UcfDNA quantification holds promise for bladder cancer diagnostics. However, current studies are limited by sample sizes and a lack of standardization. Extensive clinical trials with diverse cohorts are needed to further evaluate UcfDNA quantification, enhancing its applicability in liquid biopsy.

## 4 UcfDNA sequences as biomarkers for bladder cancer

### 4.1 Biological significance of UcfDNA mutations

Mutations and accumulations within proto-oncogenes and tumor suppressor genes in somatic cells are critical hallmarks of malignant tumors. Like lung cancer and melanoma, bladder cancer exhibits high mutation rate, emerging as a malignancy with a significant mutational burden. Analyses of bladder cancer specimens revealed 302 mutations across exons, alterations within 204 copy number segments at the genomic level, and 20 instances of genomic rearrangements (Cancer Genome Atlas Research, 2014). Recent research has identified mutations in genes such as TP53, FGFR3, PIK3CA, MLL2, CDKN1A, and ERGC2 in patients with bladder cancer. These genetic aberrations play pivotal roles in essential physiological mechanisms, including cell cycle control and programmed cell death. Notably, alterations in the tumor suppressor gene TP53 are detectable in approximately half of the cases (Cancer Genome Atlas Research, 2014). As a critical driver gene in bladder cancer, TP53 inactivation disrupts normal cellular cycle checkpoints, fostering genomic instability that promotes tumorigenesis. Additionally, TP53 expression levels correlate with bladder cancer progression and serve as significant prognostic indicators (Hollstein et al., 1991). Alterations in FGFR3 also constitute critical mutations in bladder cancer, displaying strong associations with disease stage and grade; approximately 78.1% of patients with non-invasive bladder cancer showed FGFR3 expression, whereas the expression rate dropped to 18.2% in muscle-invasive cases (Akanksha and Sandhya, 2019). Thus, mutational profiles are salient biomarkers for bladder cancer, promising tools for tumor screening, diagnosis, and ongoing assessment to elucidate tumorigenic pathways.

Obtaining tumor mutational data from tissue biopsies provides insights into cancer progression but is hindered by its invasive nature, which poses risks of complications and limits its use for long-term monitoring. Urine, readily accessible with minimal protein contamination and directly interacting with bladder cancer cells, is a preferable alternative. Numerous studies corroborate the high concordance between urine and tissue samples in terms of tumor DNA congruence (Ou et al., 2020; Zhang et al., 2021; Hirotsu et al., 2019). Parameters such as cancer cell fraction (CCF), variant allele frequency (VAF), and tumor mutation burden (TMB) consistently aligned closely between tumor DNA in urine and within tumor tissues, with less consistency observed with circulating tumor DNA (ctDNA) (Zhang et al., 2021). An innovative approach employed by Ou et al. (2020) involved both urine supernatant and sediment, yielding mutation rates and overlaps notably akin to those found in tumor tissues. This supports the potential application of this methodology for early detection and diagnosis in patients with hematuria, a common initial symptom of bladder cancer (Ou et al., 2020). Both urine supernatant and sediment can be used to assess DNA mutations in bladder cancer (Hirotsu et al., 2019). However, due to reduced contamination by immune cells and normal urothelial cells, and its more uniform composition, the urine supernatant reveals superior mutation detection capability *via* higher mutant allele frequencies (MAFs), enhancing its congruence with tumor tissue specimens (Szarvas et al., 2007; Togneri et al., 2016). Consequently, the evaluation of UcfDNA mutations holds formidable prospective clinical utility.

### 4.2 UcfDNA mutations and bladder cancer diagnosis

As previously highlighted, UcfDNA can accurately reflect the genomic landscape of the tissue of origin, indicating a significant correlation between UcfDNA and corresponding tissue samples. An extensive literature review reveals that nearly all contemporary studies have employed urine samples for the detection and diagnosis of bladder cancer, consistently achieving high sensitivity and specificity. These studies primarily utilized either the identification of individual mutations or an aggregate detection approach, focusing on genes with high mutational frequency to accomplish diagnostic objectives (Table 1).

In particular, mutations in the telomerase reverse transcriptase gene (TERT) promoter region are significant in bladder cancer, detectable in approximately 60%–70% of cases (Hurst et al., 2014) and strongly linked to carcinogenesis (Bell et al., 2016). Notably, C228T and C250T are prevalent TERT promoter mutations positioned 124 bp and 146 bp upstream of the TERT translation start site respectively (Huang et al., 2013). Studies have focused on diagnosing bladder cancer by identifying TERT promoter mutations in urine specimens (Zvereva et al., 2023). Hodonou et al. methodology presents a straightforward, non-invasive technique to detect low-abundance TERT mutations, achieving detection thresholds of 0.8% and 0.5% mutant allelic fractions (MAF) for C228T and C250T, respectively. This assay detected urothelial carcinoma with 87.1% sensitivity, 94.7% specificity, and 98.6% analytical sensitivity, demonstrating robust diagnostic potential while showing only 7.1% sensitivity in blood samples (Avogbe et al., 2019). In addition to circulating DNA fragments that are themselves in urine for detection, there is also an alternative source of DNA: sediment DNA (sDNA). Urine sDNA refers to the non-circulating DNA extracted from the precipitation of urine samples after centrifugation, which is mainly derived from cells and microorganisms in urine (Yokota et al., 1998). Because it contains many impurities, the test results have certain interference, and compared with cfDNA, sDNA acquisition process is more complicated, and the test results are more likely to be affected by operation. Separate studies confirmed comparable diagnostic merits, indicating a higher accuracy of urine sediment DNA (sDNA) over circulating free DNA (cfDNA), although cfDNA presents a greater MAF in urine from leukocyte-rich sources (Stasik et al., 2019). Beyond TERT mutations, ADGRG6 enhancer hotspot mutations manifest at a substantial rate (50%) in bladder cancer and offer diagnostic sensitivity and specificity of 83.3% and 98.4%, respectively, suggesting their utility in early screening and monitoring (Tan et al., 2023).

Integrating TERT mutation analysis with other prevalent mutations can enhance diagnostic precision. GPR126, which encodes a G-protein-coupled receptor has second-most common mutation site in urothelial bladder cancer (UBC). Analyses encompassing both GPR126 and TERT mutations across healthy controls, patients with cystitis, and patients with UBC revealed an area under the receiver operating characteristic curve (AUC) and MAF of 0.679 and 21.61, respectively, for GPR126 alone, contrast to the AUC and MAF of 0.786 and 28.29, respectively, for TERT mutations. The combined diagnostic proficiency elicited an AUC of 0.836, detecting at least one mutation in 47 of 70 urine samples, thus markedly boosting diagnostic accuracy (Jain et al., 2023). Complementary studies amalgamating TERT mutations with FGFR3 evaluations reported enhanced diagnostic accuracy, Yujiro et al. identified that combining cytology with the detection of urine TERT C228T, C250T, and FGFR3 S249C mutations yielded a sensitivity of 78.6% and a specificity of 96% for bladder cancer diagnosis (Hayashi et al., 2019). Hayashi et al. (2020) developed a swift, high-throughput detection platform for these mutations, attaining 68.9% sensitivity and 100% specificity for UBC, increasing the sensitivity up to 85.9% when combined with urine cytology.

Investigators have integrated assays for distinct genetic alterations to augment liquid applications in bladder cancer. Zhang et al. (2021) achieved a detection rate exceeding 90% for UBC by using five genes (TERT, TP53, FGFR3, PIK3CA, and ARID1A). Over 90% of patients were found to have at least one mutation within these genes, reducing sequencing efforts by four-fold (Zhang et al., 2021). Ou et al. (2020) used genetic diagnostic modeling to identify two potent combinations from a set of 48 bladder cancer-specific genes: five urinary supernatant genes (TERT, FGFR3, TP53, PIK3CA, and KRAS) and seven urinary sediment genes (TERT, FGFR3, TP53, HRAS, PIK3CA, KRAS, and ERBB2). The diagnostic accuracies of these combinations were 0.94 and 0.91 for bladder cancer, with AUCs of 0.9656 and 0.9587, respectively, which increased with advancing age (Ou et al., 2020).

### 4.3 UcfDNA expression and diagnosis of bladder cancer

Gene mutations and specific gene expression shifts serve as promising diagnostic biomarkers for BC (Table 1). Bladder cancer often initially presents through hematuria, underscoring the need for precise differentiation between BC and other hematuria-inducing disorders. Proteins activated by GTA and IQGAPs play crucial roles in cellular adhesion and migration, significantly influencing cancer progression.

In BC diagnosis, the significance of IQGAP3 increases with tumor grade, with AUC values of 0.805, 0.915, and 0.929 for G1, G2, and G3 tumor grades, respectively (Kim et al., 2018). Urinary assays of IQGAP3 facilitate the differential diagnosis between BC and hematuria, showing a Picogreen-corrected AUC of 0.91, along with sensitivity and specificity rates of 80.0% and 90.7%, respectively [46]. Furthermore, IQGAP3 can distinguish myoinvasive bladder cancer (MIBC) from non-myoinvasive bladder cancer (NMIBC), achieving sensitivity and specificity maxima of 93.8% and 80.0% for MIBC and 89.5% and 90.7% for NMIBC with PicoGreen-corrected AUCs of 0.944 and 0.87, respectively (Kim et al., 2018). Additional studies confirm that combining IQGAP3 with BMP4 or FAM07A enhances discrimination of BC from hematuric conditions (Xu et al., 2019), yielding diagnostic sensitivity and specificity scores of 71.0% and 88.6%, respectively, along with positive and negative predictive values of 90.3% and 67.2% (Xu et al., 2019).

Moreover, increased expression of topoisomerase-IIα (TopoⅡA), a subtype of DNA rotatase involved in cell cycle progression, is associated with malignant tumors (Depowski et al., 2000). In BC cases, elevated TopoⅡA expression provides superior differentiation from individuals with hematuria. Recent studies indicate that UcfDNA detection of TopoⅡA in BC, NMIBC, and MIBC achieved AUC values of 0.741, 0.701, and 0.838, respectively, highlighting its significant differentiation potential (Kim et al., 2016). Therefore, harnessing these biomarkers could substantially enhance the diagnostic accuracy of BC.

### 4.4 UcfDNA epigenetics and diagnosis of bladder cancer

DNA methylation, which involves the addition of methyl groups to the cytosine residues 5′-carbon within CpG dinucleotides, is a crucial epigenetic modification that predominantly regulates gene expression (Martisova et al., 2021). This intricate association with cancer pathogenesis is well-supported by extensive research. Alterations in DNA methylation, such as hypomethylation in retrotranspositional elements, centromeres, and oncogenes, along with hypermethylation at key regulatory loci, are common hallmarks of various cancers (Skvortsova et al., 2019; Kulis and Esteller, 2010). Analysis of cfDNA methylation signatures in body fluids presents a promising avenue for diagnosing a broad spectrum of cancers (Table 1).

The methylation detection of cfDNA at the theoretical level can be divided into two methods: bisulfite conversion-based methods and bisulfite-free methods (Luo et al., 2021). Bisulfite conversion methods are further categorized into genome-wide and site-specific assays. Representative genetic testing methods include WGBS (whole genome bisulfite sequencing) and RRBS (reduced representation bisulfite sequencing). If specific site methylation detection is needed, methods such as pyrosequencing, methylation microarray, and methylation-specific PCR (MSP) can be used. Whether for whole-genome or targeted genetic testing, the mechanism of bisulfite use remains consistent: an organism’s genome consists of only four bases—A, T, C, and G. During bisulfite treatment, unmethylated Cs are converted to Ts, while methylated Cs remain unchanged. Thus, subsequent assays can distinguish between the original and methylated sites. Methods that do not rely on bisulfite conversion can be classified into enrichment-based and restriction enzyme-based approaches.

Although cfDNA methylation detection technologies encompass various analytical methods, different methods emphasize different aspects and advantages in application. Genome-wide strategies such as microarrays and next-generation sequencing (NGS) aim to assess methylation landscapes. Quantitative techniques including bisulfite sequencing and pyrosequencing have also been optimized for this purpose (Larsen et al., 2019). Recent investigations have identified and assessed urine-derived methylation markers specific to bladder cancer (BC) by using quantitative DNA methylation-specific PCR, pyrosequencing, NGS, and digital droplet PCR methodologies (Deng et al., 2022; Tse et al., 2021; Feng and Lou, 2019). There are many methods for studying methylation, but all of them have certain limitations. In the face of specific problems, it is necessary to consider the research purpose, objective conditions, sample source and number before selecting and determining the method.

Multiplex biomarker assays have been used to enhance the sensitivity and specificity of BC diagnostics. For instance, a panel of 15 methylation markers has been successfully commercialized under the name ‘EpiCheck’ (Kohler et al., 2024; Trenti et al., 2019). Studies have also confirmed the diagnostic efficacy of TWIST1 and NID2 biomarkers, which exhibit high sensitivity and specificity for BC detection (Fantony et al., 2015; Abern et al., 2014; Renard et al., 2010). Other notable biomarker combinations include sets such as HOXA9, PCDH17, POU4F2, and ONECUT2 (wu et al., 2020), and groups comprising HOXA9, ONECUT2, PCDH17, PENK, TWIST1, VIM, and ZNF154, which achieve 90.5% sensitivity and 73.2% specificity. These are complemented by GDF15, HSPA2, TMEFF2, and VIM with a performance of 94% sensitivity and 90% specificity (Zhang et al., 2019; Costa et al., 2010). These findings underscore the potential of UcfDNA as a diagnostic biomarker for BC.

Ample research indicates the growing applicability of cfDNA methylation in cancer screening (Liu et al., 2020; Klein et al., 2021). Beyond cancer detection, cfDNA methylation can identify the tissue of origin of tumor genes and show increased detection rates as cancer progresses. Gene panels involving SFRP1, SOX9, FHIT, CDH1, PMF1, RUNX3, LAMC2, RASSF1A, TWIST1, NID2, and RBBP8 have demonstrated relevance in BC pathophysiology, making these methylated targets predictive indicators of patient prognosis and treatment outcomes (Mijnes et al., 2018; Mohanad et al., 2022; Kandimalla et al., 2013). Moreover, these biomarkers have shown significant correlations with BC staging. For example, DMRTA2 has emerged as a potential marker for early-stage BC detection, exhibiting high sensitivity for stages T1 (90.4%) and T2 (95.0%), and an overall sensitivity of 82.9% (Deng et al., 2022). Innovative advancements, such as refined urine DNA methylation panels incorporating biomarkers like PCDH17, POU4F2, and PENK, have achieved impressive detection accuracy with 97% sensitivity and 87% specificity (Harsanyi et al., 2022). Meanwhile, methodologies such as cfMethyl-Seq pioneered by Stackopol et al. and Zeng et al., have enhanced CpG island discernibility across diverse cancer types, with sensitivity ranging from 75.9% to 92.3% in plasma sample analyses (Stackpole et al., 2022).

## 5 Advanced techniques for UcfDNA in bladder cancer diagnosis

Several widely employed technologies for UcfDNA detection, such as PCR and next-generation sequencing (NGS), have been discussed. The landscape of detection methods is evolving rapidly, with an increasing array of innovative techniques being developed.

### 5.1 Lateral flow assay

The Lateral Flow Assay (LFA) is a paper-based diagnostic platform that facilitates the detection and quantification of analytes in complex mixtures. Recognized as an effective tool for qualitative, semiquantitative, or quantitative biomarker analysis, LFAs are distinguished by their low detection costs, rapid response times, and simplicity of operation. These attributes make them invaluable for a wide range of applications, including infectious disease screening, food safety assessments, and environmental monitoring (Huang et al., 2016).

**FIGURE 2:**
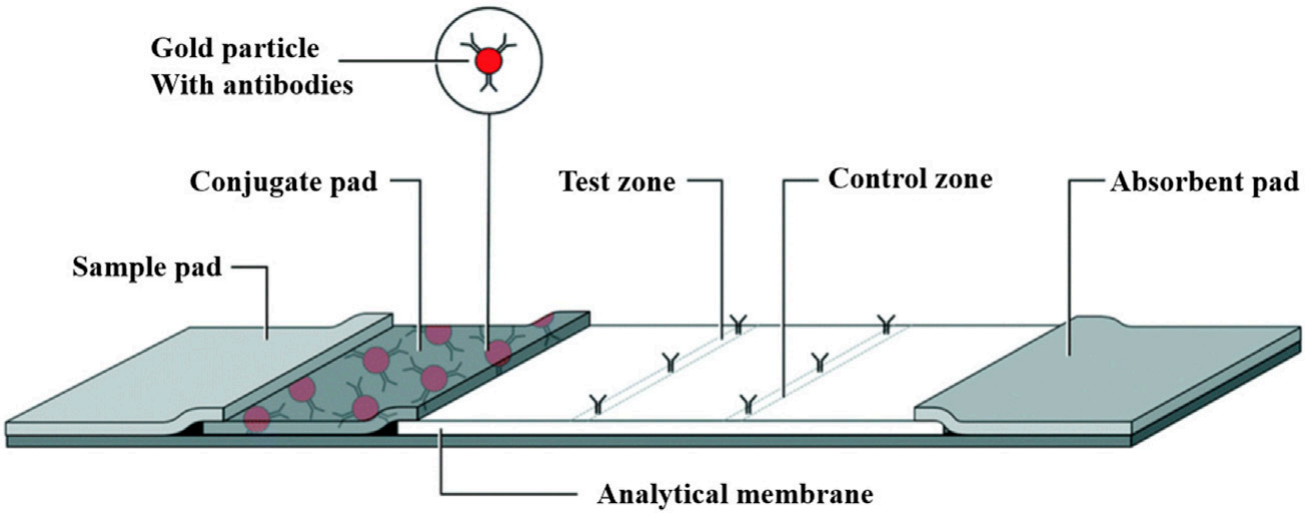
Lateral flow assay schematic: The LFA assay consists of a sample pad, a conjugate pad, and an analytical membrane containing immobilized capture reagents. The sample is added to the sample pad and transferred by capillary action to the conjugate pad and binds to the detection reagent, which is then transferred to the test zone at the analytical membrane to generate a detectable light signal, the intensity of which correlates with the concentration of the detection reagent that binds to the sample. The excess liquid is drawn into the absorbent pad at the back [The figure was modified from that reported in its original publication (Bishop et al., 2019)].

Comprising four primary components, the LFA includes a sample pad for specimen application, a conjugate pad that introduces labeled biometric molecules, a nitrocellulose membrane critical for assay sensitivity, and an absorbent pad that captures excess samples (Bishop et al., 2019) (Figure 2). LFAs use two principal methodologies: sandwich (sLFA) and competitive (cLFA). LFAs are primarily used for analytes with multiple epitopes, such as proteins, antibodies, cells, and bacteria, while cLFA are suitable for small molecules or haptens with a single epitope. Technological advances have expanded LFA labeling options beyond using gold nanoparticles and polystyrene microbeads for qualitative results, incorporating multiple test lines or points on a single strip to facilitate analysis.

Significantly, LFAs can detect antigens and antibodies and be integrated with molecular amplification techniques, such as loop-mediated isothermal amplification (LAMP) and PCR, for DNA detection. The existing literature highlights the successful identification of nucleic acids in urine samples. Jin et al. introduced an MCL-PRPA-HLFIA cascade system that leveraged RPA enhanced by PEG 200 and HRP-driven lateral chromatography immunoassays. This refined Chelex-100-based lysis method enables swift DNA extraction from urine, streamlining the detection of pan-drug-resistance genes associated with tract infections without the need for high-speed centrifugation or pre-incubation of samples (Tao et al., 2023). In parallel, Cheng et al. detailed a dual-isothermal cascade approach using a lateral flow peptide-nucleic acid biosensor, advancing the utility of this methodology in bladder cancer diagnostics through microRNA detection in urine samples. Despite the current limitations in the application of LFAs for UcfDNA detection in bladder cancer, anticipation of broader clinical adoption remains robust (Cheng et al., 2017).

This ongoing evolution underscores the substantial potential of LFA technologies to contribute significantly to biomedical diagnostics and promises more focused and efficient-patient care modalities in diverse clinical settings.

### 5.2 Microfluidics

Microfluidics, often referred to as “lab-on-a-chip” (LOC) technology, enables precise manipulation of fluids on the micron scale. Single-molecule-level detection can be achieved by integrating fundamental biochemical processes, such as sample preparation, reaction, separation, and detection, within a microchip. This technology offers automated operation, cost-efficiency, and high-throughput capabilities (Xiong et al., 2014). By requiring minimal sample volumes, microfluidic devices can swiftly and economically conduct complex biochemical assays, proving instrumental in various biomedical applications, such as disease marker identification and clinical diagnostics. Additionally, their compact scale and automation potential minimize the risk of sample contamination during transfer, making them ideal for sustained monitoring (Shen et al., 2010). Two principal strategies exist for DNA extraction and purification in microfluidics: solid-phase methods involving surface-functionalized platforms or immobilized magnetic beads, and liquid-phase techniques utilizing chemical agents or leveraging electrophoresis (EP) and dielectrophoresis (DEP) to drive negative DNA migration (Sun et al., 2018). Microfluidics show significant promise for liquid biopsy applications, particularly in the context of UcfDNA analysis.

**FIGURE 3:**
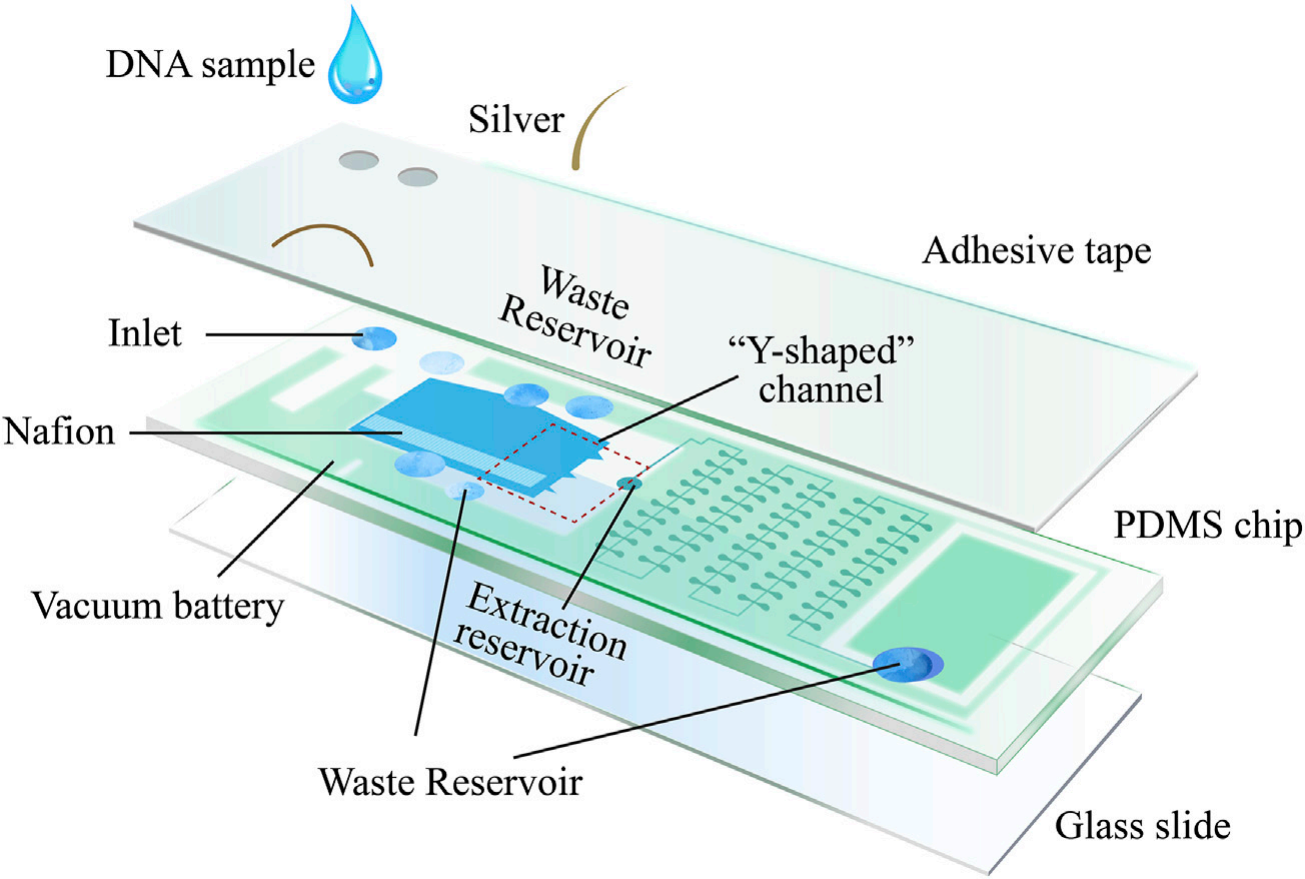
Microplatform device schematic: the device consists of three main compartments: the enrichment region, the amplification region, and the vacuum battery system. The vacuum battery system is highly permeable to PDMS, which provides sample propulsion by gassing between the vacuum channel and the microchannel. After the sample enters the enrichment region, the negatively charged nucleic acids are electrodynamically captured by the vertically applied electric field and cation selectively permeable membrane as well as the horizontal driving force, creating an ion-enriched zone. Subsequently, the enriched nucleic acids are transported by the horizontal driving force from the “Y-shaped” channel to the downstream amplification, where they are divided into different wells and amplified isothermally. The fluorescence intensity within the sample is analyzed according to the number of fluorescence-positive wells and the fluorescence intensity in the positive wells after amplification [The figure was modified from that reported in its original publication (Wang et al., 2024)].

Recent research has introduced an innovative, unpowered multifunctional microplatform that combines DNA enrichment *via* field-flow fractionation with induced continuous polarity (FF-ICP) and loop-mediated isothermal amplification (LAMP). This platform facilitates the isolation, enhancement, amplification, and detection of cfDNA from diverse clinical samples, including serum, urine, and fecal matter (Wang et al., 2024) (Figure 3). The system uses vertically applied electric fields and cation-selective membranes to create continuous forces that drive negatively charged biomolecules downward by electroosmosis, while simultaneously enhancing upward electrophoretic motion, resulting in electrically-concentrated molecular zones. Horizontal movement is achieved using a self-sustaining vacuum cell mechanism. Air dispersion through the PDMS walls along with pre-degassed PDMS, drives the flow of liquid samples for collection. To seamlessly transition from the initial DNA enrichment stage to subsequent amplification, a “Y” extraction channel is strategically positioned relative to the enrichment locus within the device. The channel directs the enriched DNA to the processing chambers, where lyophilized components necessary for LAMP reactions are stored. After enrichment, the DNA is mixed with these reagents, facilitated by a microarray setup that allows for concurrent nucleic acid concentration quantification following amplification. This platform enhances the rapidity and efficacy of cfDNA enrichment and purification from various clinical specimens and incorporates on-chip isothermal amplification, thereby improving cfDNA detection sensitivity and effectively reducing the occurrence of false negatives due to low cfDNA presence in serum or urine-based analyses. It represents an all-in-one solution for cfDNA isolation, enrichment, amplification, and readout from clinical samples.

Advancements extend beyond this platform to standalone and fully integrated fluidic systems using adjunct analyzers, for multiplexed bacterial DNA detection in urine specimens. These include magnetic silica bead-based DNA extraction, elution, and amplified sensitivity for loop-mediated amplification (LAMP) (Li et al., 2020). Despite the early stages of microfluidic implementation for UcfDNA detection in bladder cancer, substantial progress has been made toward the efficient isolation of cfDNA from bodily fluids. Li et al. (2020) introduced an innovative pressure and immiscible fluid extraction (PIBEX)-based microfluidic chip, that does not require centrifugal force and can extract cfDNA from plasma under vacuum-induced pressure using an immiscible solvent. This process, lasting only 15 min, rivals the purification efficiency and purity of popular commercial kits such as QIAamp (Lee et al., 2020). Balakrishnan et al. (2021) employed superparamagnetic (SPM) bead particles for cfDNA extraction from blood specimens, achieving sensitivity and specificity rates of 65.57% and 95.38%, respectively, for early-stage cancer detection. Guan et al. (2017) incorporated PCR pre-amplification into a microfluidic workflow tailored for multiplex PCR sequencing, achieving an overall detection sensitivity and specificity of 92% and 100% for cfDNA mutations. Baudrey et al. (2022) utilized digital drop PCR (ddPCR), which segments DNA molecules into discrete partitions before amplification, reducing competition among similar DNA templates and offering a novel technique for highly sensitive absolute DNA quantification. Additionally, Zhao developed the first multiple digital methylation-specific PCR (mdMSP) system, enabling the simultaneous assessment of four methylation biomarkers in cfDNA, demonstrating robust clinical utility for non-small cell lung cancer (NSCLC) liquid biopsies (Zhao et al., 2023). Although microfluidic applications for bladder cancer UcfDNA remain exploratory, existing methodologies for the detection of urinary bacterial DNA and blood cfDNA hold considerable promise and pave the way for further research.

### 5.3 Biosensors

**FIGURE 4:**
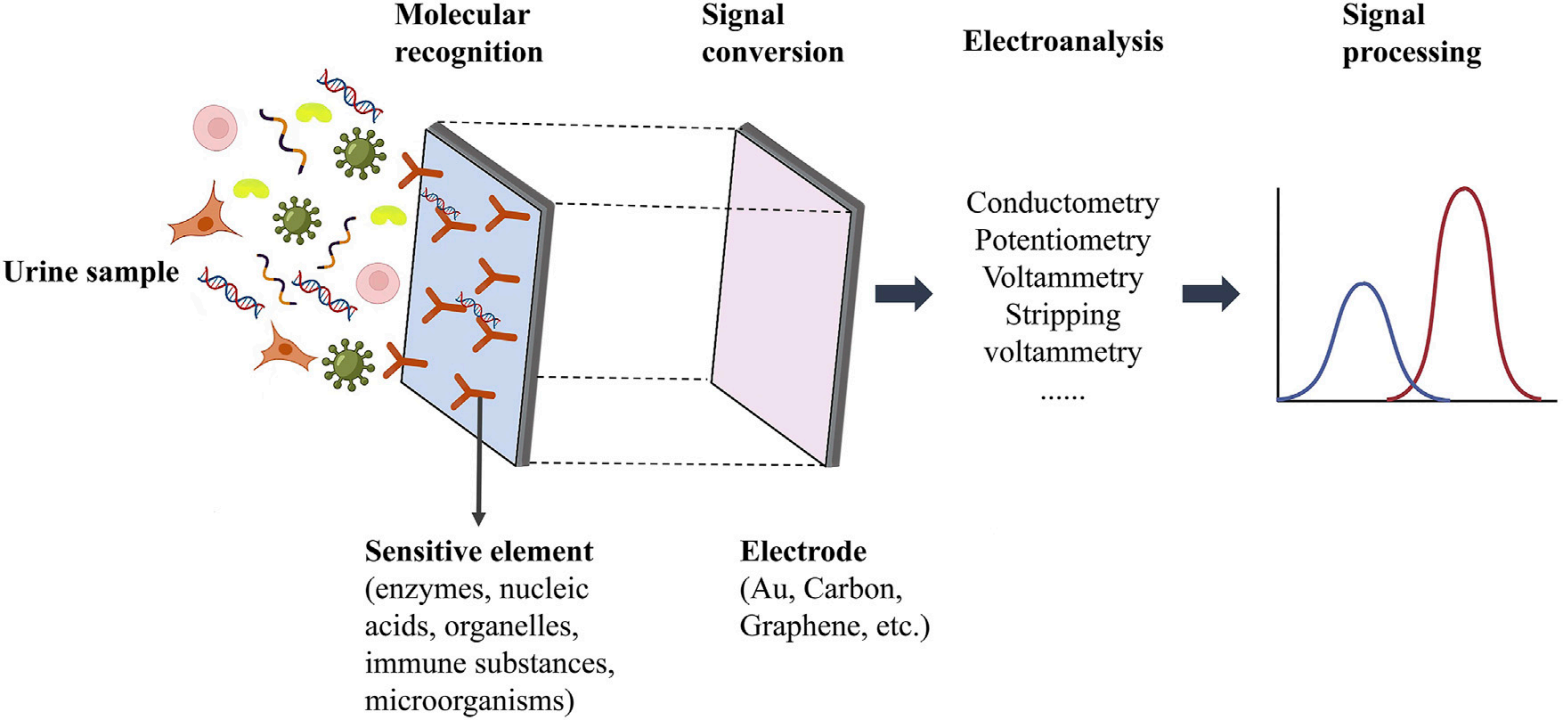
Schematic diagram of cfDNA electrochemical biosensor detection system [The figure was modified from that reported in its original publication (Wang et al., 2022)].

Biosensors, that combine biological recognition components (such as DNA, RNA, antibodies, and enzymes) with signal-conversion mechanisms are emerging as prominent tools in clinical diagnostics. These biosensors can be categorized into electrochemical, optical, magnetic, and thermal variants based on their signal transduction methodologies. Electrochemical biosensors, known for their high sensitivity, specificity, portability, and quick response time, offer significant advantages in for cfDNA detection (Sharifi et al., 2020). Typically, these biosensors consist of a biomolecular recognition element, a conductive electrode serving as the signal converter, and a signal transduction mechanism (Wang et al., 2022) (Figure 4).

For the selective identification and capture of cfDNA, DNA sensors frequently use peptide nucleic acid (PNA) probes or DNA probes. The literature documents multiple methodologies that facilitate nucleic acid detection in bladder cancer urine samples (Cheng et al., 2023; Guo et al., 2022). Cheng introduced an innovation involving tetrahedron-supported CRISPR/Cas13 cleavage enabling direct electrochemical detection of circRNAs in bladder cancer urine, with detection possible in volumes as small as 25 μL and in under 10 min (Cheng et al., 2023). Similarly, Smith developed an approach for the electrochemical detection of low concentrations of human urinary miRNA using *via* a complementary DNA-modified glassy carbon electrode (Smith et al., 2017).

Moreover, gene mutation detection using electrochemical biosensors is feasible, as demonstrated by techniques such as combination probes (Das et al., 2018), dual-enzyme-assisted multiple amplification strategies (Wang et al., 2018), and conductive metal-organic frameworks (Liu et al., 2022). In ovarian cancer diagnostics, targeting a 110-nucleotide DNA sequence with single-point methylation allows biosensors to achieve a broad dynamic range of target DNA methylation and low detection limits, resulting in rapid ovarian cancer diagnosis within 35 min (Chen et al., 2022). Consequently, cfDNA-based liquid biopsy presents a novel and promising method for bladder cancer diagnosis.

## 6 Advanced bioactive materials for UcfDNA in bladder cancer diagnosis

An increasing number of research teams are developing bioactive materials capable of adsorbing and targeting UcfDNA. These advancements are crucial for the enrichment and isolation of unencapsulated cfDNA and offer new therapeutic avenues for cfDNA-related disorders (Wu et al., 2023).

### 6.1 Polyethyleneimine-coupled magnetic polypyrrole nanowires

**FIGURE 5:**
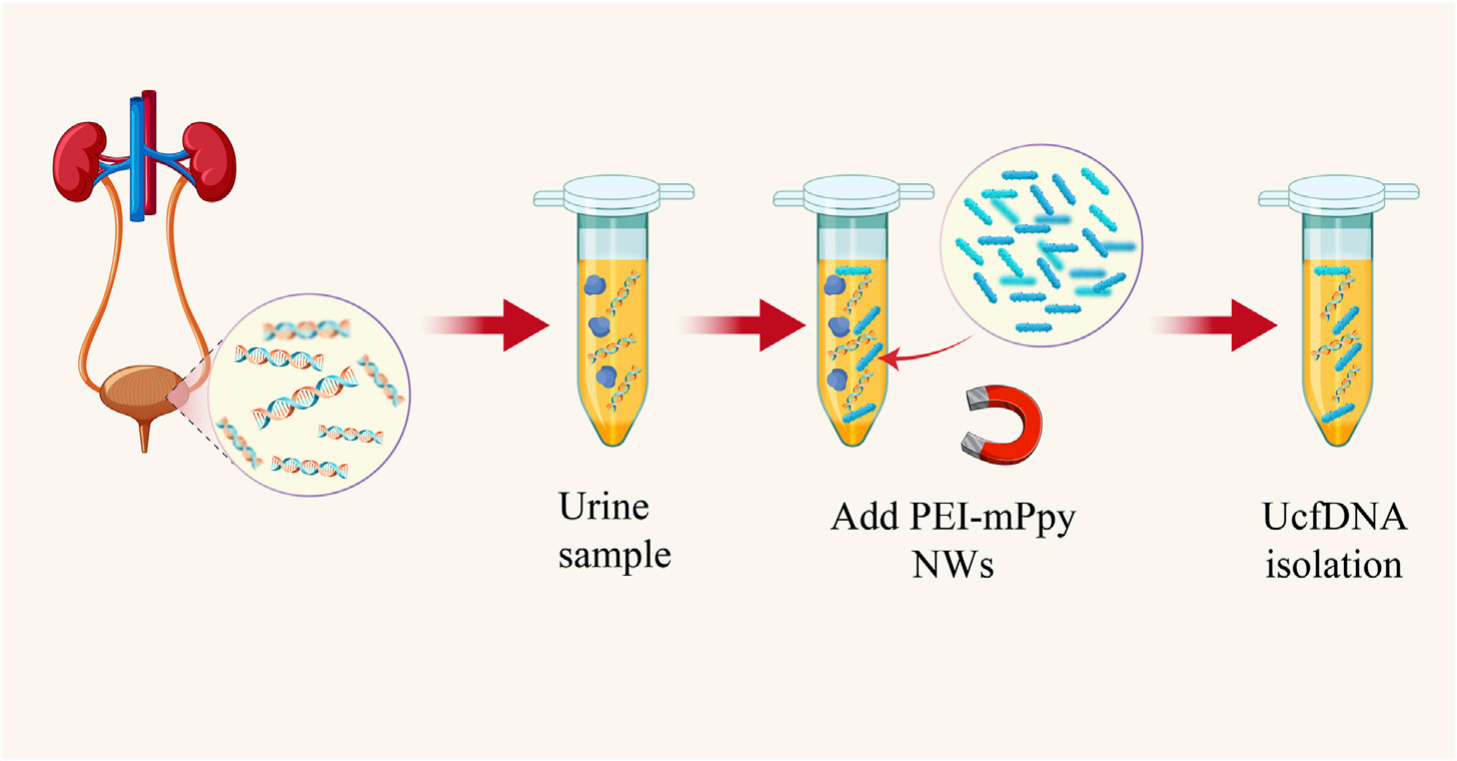
Schematic diagram of highly efficient isolation of UcfDNA [The figure was modified from that reported in its original publication (Lee et al., 2018)].

A promising strategy involves using polyethyleneimine-coupled magnetic polypyrrole nanowires (PEI-mPpy NWs) to facilitate the rapid and efficient separation of cell-free cfDNA (cfDNA) from urine samples. This technique yields high purity and a significant considerable concentration of cfDNA. Additionally, this methodology employs horseradish peroxidase (HRP)-conjugated and streptavidin-tagged polypyrrole nanoparticles (HRP/st-labeled NPs), to enhance the colorimetric detection signal for targeted cfDNA extraction and quantitation (Lee et al., 2018) (Figure 5).

Due to their large surface-area-to-volume ratio and pencil-like structure, PEI-mPpy NWs exhibit directional movement within a magnetic field. These attributes allow unencumbered movement within the complex urinary matrix, enabling selective binding to cfDNA. The affinity of the nanowires for cfDNA is determined by the properties of polyethyleneimine (PEI), a multivalent amino-rich cationic compound adept at adsorbing anionic cfDNA. Thus, the hybrid structure of PEI-mPpy NWs not only binds to the target cfDNA but also facilitates its manipulation and partitioning from other urine constituents in a magnetic field.

After isolation, a customized set of biotinylated capture probes (CPs) and detection probes (DPs), dissolved in a solution containing a 1 mM colorimetric TMB substrate and a 0.5 mM H_2_O_2_ catalyst, are introduced to the system, targeting the corresponding DNAs attached to the nanowires. Following hybridization, the addition of HRP and streptavidin anti-biotin protein (st)-labeled Ppy nanoparticles (HRP/st NPs) allows for specific recognition, significantly enhancing the H_2_O_2_-induced catalytic oxidation of TMB. This process results in a pronounced colorimetric shift, contingent on the presence of the target DNA. Empirical evidence indicates a direct correlation between the absorbance of oxidized TMB at 650 nm and the target DNA concentration, enabling the quantification of cfDNA *via* colorimetric analysis. Collectively, such an innovative approach utilizes bioactive PEI-mPpy NWs for the isolation and enrichment of urinary cfDNA, complemented by amplified colorimetric signaling mediated by supplementary probes and HRP/st NPs for targeted cfDNA identification and isolation.

### 6.2 ZnO nanowires

**FIGURE 6:**
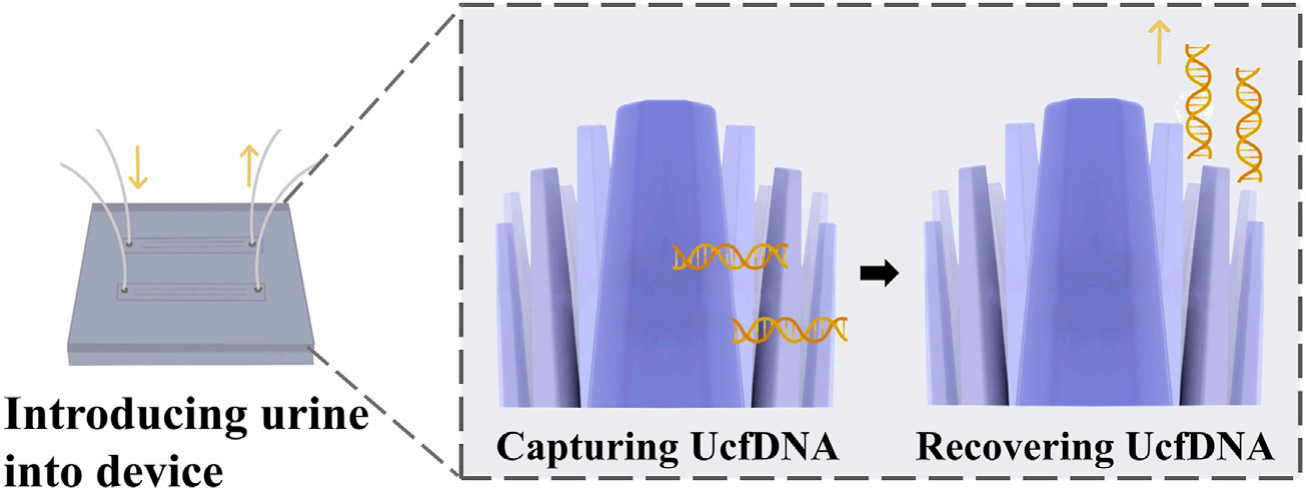
Nanowire-based cfDNA capture, cfDNA is captured by multi-point hydrogen bonding and surface charge on ZnO nanowires [The figure was modified from that reported in its original publication (Takahashi et al., 2023)].

Takahashi et al. developed a method using ZnO nanowires to capture cfDNA from urine through hydrogen bonding (Takahashi et al., 2023) (Figure 6). The principle behind this method is that the strength of the hydrogen bonds between DNA and nanowires is intermediate between the intermolecular hydrogen bonds of DNA and those formed by hydration. This allows UcfDNA to be adsorbed onto the nanowires through hydrogen bond formation. After adsorption, the cfDNA is captured by removing the ZnO nanowire device. Ethylenediaminetetraacetic acid (EDTA), is then introduced, as it has a stronger affinity for nanowires than DNA. EDTA competitively binds to ZnO, releasing the cfDNA and thereby achieving its separation. Compared to conventional methods, this technique offers higher separation efficiency, shorter processing times, and requires smaller sample volumes. Commercial kits often fail to separate UcfDNA with high yields, hindering further nucleic acid analysis. However, this nanomaterial-based separation method enables both the capture and release of cfDNA from clinical samples, as well as the identification of tumor molecular subtypes, thereby contributing to the diagnosis of bladder cancer.

### 6.3 Zn-DPA-modified slide

**FIGURE 7:**
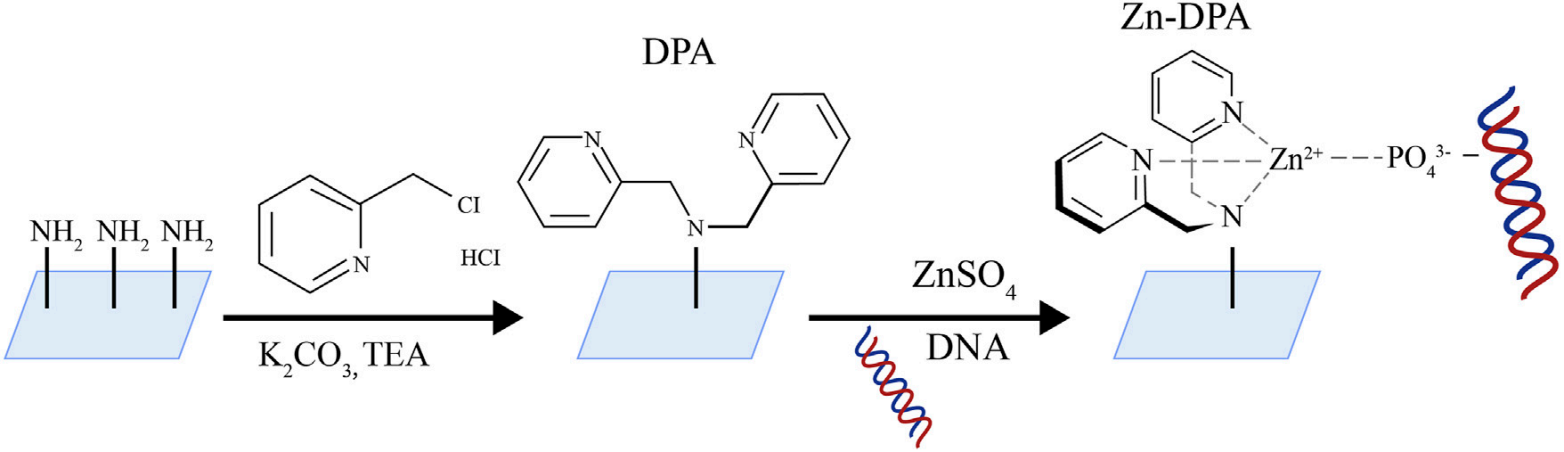
Schematic diagram of Zn-DPA modified slide [The figure was modified from that reported in its original publication (Wu et al., 2024)].

Wu et al. (2024) developed a method for the portable and visual quantification of UcfDNA *via* a smartphone-based colorimetric biosensor (Figure 7). This method involves enriching of UcfDNA using slides coated with zinc ion-dipyridylamino complexes, followed by labeling with PicoGreen (PG). DNA-PG complexes are formed through photocatalysis resulting in a visible color change. This color change is then quantitatively analyzed using a smartphone-assisted colorimetric biosensor platform. The core material used of this method is an innovative slide modified with zinc ion-dipyridylamino (Zn-DPA), which exhibits a strong affinity for the phosphate groups of negatively charged DNA, facilitating efficient DNA enrichment.

The core material of this method is a Zn-DPA-modified glass slide. The team employed a series of clever substitution reactions to produce the slide: initially, the glass surface was modified with polyethyleneimine (PEI) to introduce abundant amino groups. Subsequently, zinc-dimethylpyridylamine (Zn-DPA) was further modified onto the glass surface. The free amine groups in PEI on the glass slide underwent nucleophilic substitution reactions with two molecules of 2-chloromethylpyridine, forming dimethylpyridylamine (DPA) structures with metal chelating functionality. Afterwards, the DPA-modified glass slide was co-incubated with zinc sulfate heptahydrate solution, allowing zinc ions to coordinate with the pyridylamine groups on DPA, forming the zinc-dimethylpyridylamine (Zn-DPA) modified glass slide. PEI facilitated the subsequent Zn-DPA modification on the glass slide by providing numerous active sites. Dimethylpyridylamine played a role in forming stable complexes with zinc ions. The open sites formed by DPA and Zn^2+ in the Zn-DPA complex effectively coordinated with oxygen anions in phosphate groups, exhibiting strong adsorption towards cfDNA rich in phosphate groups. This design based on coordination chemistry principles enables the Zn-DPA-modified glass slide to efficiently adsorb and enrich cfDNA. This method showed superior linearity (*R* ^2^ > 0.95) compared to a two-stage commercial kit, aiding in the differentiation between healthy individuals and those with urological disorders. Additionally, the colorimetric biosensor platform can assess the severity of urological disorders using either unadorned dyes or color cards. The photocatalytic strategy for cfDNA enrichment and amplification operates synergistically, enabling sensitive and visible DNA detection. In this context, the current work represents a swift, economical, and portable approach for the quantitative assessment of UcfDNA.

## 7 Conclusion

Urinary cell-free DNA (UcfDNA) offers significant advantages as a non-invasive and easily obtainable biomarker, presenting extensive applications in urologic pathology. Current detection strategies for UcfDNA encompass both quantitative and qualitative assessments, mutation screening, gene expression analysis, and epigenetic evaluation. These diverse methodologies demonstrate substantial sensitivity and specificity for diagnosing bladder cancer, suggesting a promising potential to replace conventional tissue biopsies and urinary cytology.

Despite these advancements, the majority of studies on bladder cancer diagnosis using UcfDNA have been conducted in a laboratory setting. Further research is necessary to validate their clinical utility and undergo standardized testing. Additionally, existing UcfDNA detection methods are often technologically complex and costly, posing a significant barrier to their clinical application. Some new detection technologies, however, offer automation, low cost, high throughput, and rapid response, such as LFA, microfluidics, and biosensors. These technologies have been demonstrated to be applicable to the detection of cfDNA in body fluids, possessing immense potential in liquid biopsy. Nevertheless, research on cfDNA from urine sources remains relatively limited, hindering its application in bladder cancer diagnosis and postoperative monitoring. In the future, more research is required to explore and validate the application of these new technologies in bladder cancer. Furthermore, novel materials have been proven to purify UcfDNA from urine at high concentrations, such as PEI-mPpy NWs, ZnO nanowires, and Zn-DPA. These materials provide new methods for UcfDNA extraction and require further practice to integrate them with different detection methods, thereby truly realizing their clinical application.
